# The Functional Integration in the Sensory-Motor System Predicts Aging in Healthy Older Adults

**DOI:** 10.3389/fnagi.2016.00306

**Published:** 2017-01-05

**Authors:** Hui He, Cheng Luo, Xin Chang, Yan Shan, Weifang Cao, Jinnan Gong, Benjamin Klugah-Brown, Maria A. Bobes, Bharat Biswal, Dezhong Yao

**Affiliations:** ^1^The Key Laboratory for NeuroInformation of Ministry of Education, Center for Information in BioMedicine, High-Field Magnetic Resonance Brain Imaging Key Laboratory of Sichuan Province, School of Life Science and Technology, University of Electronic Science and Technology of ChinaChengdu, China; ^2^Department of Biological Psychiatry, Cuban Neuroscience CenterLa Habana, Cuba; ^3^Department of Biomedical Engineering, New Jersey Institute of Technology, University Heights, NewarkNJ, USA

**Keywords:** aging, resting state fMRI, functional connectivity, sensory-motor system, machine learning

## Abstract

Healthy aging is typically accompanied by a decrease in the motor capacity. Although the disrupted neural representations and performance of movement have been observed in older age in previous studies, the relationship between the functional integration of sensory-motor (SM) system and aging could be further investigated. In this study, we examine the impact of healthy aging on the resting-state functional connectivity (rsFC) of the SM system, and investigate as to how aging is affecting the rsFC in SM network. The SM network was identified and evaluated in 52 healthy older adults and 51 younger adults using two common data analytic approaches: independent component analysis and seed-based functional connectivity (seed at bilateral M1 and S1). We then evaluated whether the altered rsFC of the SM network could delineate trajectories of the age of older adults using a machine learning methodology. Compared with the younger adults, the older demonstrated reduced functional integration with increasing age in the mid-posterior insula of SM network and increased rsFC among the sensorimotor cortex. Moreover, the reduction in the rsFC of mid-posterior insula is associated with the age of older adults. Critically, the analysis based on two-aspect connectivity-based prediction frameworks revealed that the age of older adults could be reliably predicted by this reduced rsFC. These findings further indicated that healthy aging has a marked influence on the SM system that would be associated with a reorganization of SM system with aging. Our findings provide further insight into changes in sensorimotor function in the aging brain.

## Introduction

Healthy aging is typically accompanied by functional and structural changes in the brain. Decrease in motor performance and movement coordination is one of the most consistent findings in older adults (Seidler et al., 2010; Allen et al., 2011; Hoffstaedter et al., 2014), and is an important aspect of physiological aging. The general slowing of movements accompanied with aging has been observed in previous studies (Birren and Fisher, 1995). The primary sensory-motor (SM) system plays a critical role for somesthesia and movement generation. Accumulating evidence suggests that age related resting-state functional connectivity (rsFC) decreases in the SM network (Tomasi and Volkow, 2012). Furthermore, several prior studies have found both motor performance and age to be associated with connectivity strength in older adults, suggesting that it may serve as a biomarker of brain health and functional performance (Langan et al., 2010; Seidler et al., 2015).

Age-related functional and structural declines in the SM system and their possible impact on sensorimotor performance are quite well documented (Seidler et al., 2010). During an isometric handgrip task, previous study demonstrated that activity in the contralateral primary motor cortex, cingulate sulcus and a premotor cortex co-varied positively with increasing force output in younger adults, but was less prominent in older adults (Ward et al., 2008). These findings possibly indicate a reduced ability to modulate activity in appropriate motor networks in older adults (Seidler et al., 2010). In recent studies, with increasing age, the reduced rsFC between the mid-posterior insula and subthalamic nucleus (Mathys et al., 2014), as well as SMA and central insula (Hoffstaedter et al., 2014) that plays an important role in sensorimotor integration processing (Deen et al., 2011; Chang et al., 2013; Uddin, 2015), have been thought to be associated with the age of older adults. Furthermore, Seidler et al. (2015) found that greater rsFC of SM system was linked to better motor performance in healthy older adults. It was thus concluded that changes in the resting-state of the SM system might contribute to the sensorimotor performance observed in older adults (Seidler et al., 2015).

Several previous structural and functional studies using magnetic resonance imaging (MRI) scans have also shown developmental trajectories in brain maturation and aging (Dosenbach et al., 2010; Rodrigue and Kennedy, 2011; Cao et al., 2014, 2016; Khundrakpam et al., 2015). Khundrakpam et al. (2015) found that the top predictors of brain maturity were found in highly localized sensorimotor (precentral and postcentral gyrus, insula) and association areas (including middle and superior frontal gyrus) in normally growing children and adolescents. Similarly, Dosenbach et al. (2010) reported that rsFC of SM network contributed in estimating chronological age in the typically developing volunteers. However, fewer studies have examined the predictive model of chronological age in healthy older adults (Qiu et al., 2015). Aging of the brain’s structure over the course of the adult lifespan has been characterized by decreased gray matter volume (GMV) in prefrontal cortex and primary sensory cortices (Rodrigue and Kennedy, 2011). Changes in the resting-state of the SM system might contribute to estimate the age of older adults (Qiu et al., 2015). Based on the existing literature, it is important to ascertain the intrinsic rsFC patterns of the SM system in older adults. Thus, we hypothesized that participants with advanced age would demonstrate abnormal SM system connectivity; moreover, we further speculated that the age of older individuals would be predicted by decreased intrinsic functional connectivity of the SM system.

In the present study, to validate our hypothesis, a cohort of healthy aging subjects was recruited in resting state fMRI test. First, we analyzed resting state fMRI data to evaluate the impact of healthy aging on the primary sensorimotor system from global (independent component analysis, ICA) and local (seed-based functional connection analysis) aspects. In addition, we used machine learning approaches from two-aspect connectome-based prediction frameworks contain multivariate pattern analysis (MVPA) and univariate pattern analysis (UVPA) tools to examine brain-based predictors of individual differences in the age of older adults.

## Materials and Methods

### Subjects

Two groups of test subjects were recruited for this study, including 68 healthy right-handed older adults [age (mean ± SD): 63.5 ± 6.5 years (51–78 years); the years of education: 9.9 ± 3.2 years (6–14 years); *n* = 37 females] and fifty-seven healthy right-handed younger adults [age: 20.5 ± 2.2 years (18–26 years); the years of education: 13.9 ± 1.2 years (13–16 years); *n* = 28 females]. None of the participants had a history of substance abuse, neurological or psychiatric disorders. All older subjects were assessed using neuropsychological and health test batteries including the health scale named Chinese 36-item short-form health survey (SF-36), which consisted of 36 items and tapped eight health concepts (Li et al., 2003), and the neuropsychological test named Montreal Cognitive Assessment (MoCA), which was specifically developed to screen for mild cognitive impairment (Nasreddine et al., 2005). Subjects with poor performance on the SF-36 and low MoCA score (<25) were excluded from this study. All the participants gave informed consent and the research protocol was approved by the Ethics Committee of the University of Electronic Science and Technology of China. All subjects were financially compensated for their time.

### Imaging Data Acquisition

Images were acquired on a 3T MRI scanner (GE DISCOVERY MR750) at the MRI Research Center of University of Electronic Science and Technology of China. During scanning, foam padding and ear plugs were used to reduce head motion and scanning noise, respectively. Resting state functional MRI data were acquired using gradient-echo echo planar imaging sequences (repetition time [TR] = 2000 ms, echo time [TE] = 30 ms, flip angle [FA] = 90°, matrix = 64 × 64, field of view [FOV] = 24 cm × 24 cm, slice thickness/gap = 4 mm/0.4 mm), with an eight channel-phased array head coil. A 510-second resting state scan (yielding 255 volumes) was collected from each of the subjects. Subsequently, high-resolution T1-weighted images were acquired using a 3- dimensional fast spoiled gradient echo (T1-3D FSPGR) sequence (TR = 6.008 msec, FA = 9°, matrix = 256 × 256, FOV = 25.6 cm × 25.6 cm, slice thickness = 1 mm, no gap, 152 slices). During resting-state fMRI, all subjects were instructed to have their eyes-closed and to move as little as possible without falling asleep.

### fMRI Preprocessing

Data preprocessing was performed using SPM8^1^ (Statistical Parametric Mapping). The first five volumes were discarded for the magnetization equilibrium from all fMRI scans. A series of preprocessing steps was performed for each subject: (1) slice timing correction; (2) head motion correction; (3) normalization: in detail, the mean images resulted from the motion correction step were segmented into gray matter, white matter, and cerebrospinal fluid using the “unified segmentation” (Ashburner and Friston, 2005). Then, we could get the resulting parameters of a discrete cosine transformation, which defines the deformation field to move subject data into Montreal Neurological Institute (MNI) space. The deformation was subsequently applied to transform each echo planar imaging volume into the MNI single-subject space. The resulted images were resampled at 3 mm isotropic voxel size; (4) images were smoothed by an 8-mm full width at half maximum Gaussian; (5) temporal filtering was performed in band-pass 0.01–0.08 Hz (Fox et al., 2005); (6) nuisance signals were regressed out, including white matter, cerebrospinal fluid and global signal, and six motion parameters. Subjects who had a maximum translation in any of the cardinal directions larger than 1 mm or a maximum rotation larger than 1° were excluded from subsequent analysis. In addition, we also assessed framewise displacement translation (*FD_translation_*) and framewise displacement rotation (*FD_rotation_*) in both groups using the following formula:

$$\begin{array}{c} FD_{translation/rotation}=\frac{1}{M\;-\;1}\sum_{i\;=\;2}^{M}\sqrt{|\Delta \;d_{x_{i}}{|}^{2}\;+\;|\Delta \;d_{y_{i}}{|}^{2}\;+\;|\Delta \;d_{z_{i}}{|}^{2}} \end{array}$$

where *M* is the length of the time courses (*M* = 250 in this study), x_i_, y_i_, and z_i_ are translations/rotations at the *i*th time point in the *x, y*, and *z* directions, respectively, ΔD_X_i__ = X_i_ - X_i-1_, and similar for D_yi_ and D_zi_.

### GMV Calculation

Controlling functional connectivity maps by adding the GMV as a covariate in the rsFC analysis (Damoiseaux et al., 2008) could increase the reliability of resting state fMRI studies and indicate whether changes in rsFC maps are associated with brain atrophy. To obtain the GMV, T1-weighted images were processed using SPM8 toolbox with spatial normalization to MNI-space using a diffeomorphic anatomical registration through exponentiated lie algebra (DARTEL), and segmentation into gray matter, white matter and cerebrospinal fluid. The segmented gray matter images were modulated using nonlinear deformation. Individual GMV of the whole brain was calculated by setting a threshold at a probability of 80%.

### Sensory-Motor Connectivity Analysis

The SM system is a common resting state network reported in previous studies. In general, there are two common approaches to identify this system: a data-driven method and a hypothesis-driven method. The ICA is selected for the former; the typical choice for the latter is the seed-based rsFC analysis with seed at the motor and somatosensory cortex. These two methods were adopted in this study to evaluate the rsFC of the sensorimotor system in younger and older adult subjects.

First, the data-driven method, ICA, was performed in two groups. Group spatial ICA (Calhoun et al., 2001b) was conducted using GIFT software^2^ (Version 2.0). We used minimum description length (MDL) (Li et al., 2007) to validate the number of ICA components. For computational feasibility, principal component analysis was used to reduce data dimensionality. The infomax algorithm was repeated 30 times in ICASSO^3^ and the resulting components were clustered to estimate the reliability of the decomposition. Finally, spatial maps and time courses were reconstructed for each subject using the group ICA (GICA) back-reconstruction method based on principal component analysis compression and projection (Calhoun et al., 2001a). The sensorimotor network component were visually inspected and selected.

The resting state networks comprising the primary motor somatosensory cortices were estimated using a seed-based analysis. Based on our previous research work (Luo et al., 2012), four nearly spherical regions (radius 6 mm) were selected from the bilateral primary motor cortex (right M1, MNI coordinates [47–15 57]; left M1, MNI coordinates [–44–15 58]) for the motor network, and the bilateral primary somatosensory cortex (right S1, MNI coordinates [53–26 59]; left S1, MNI coordinates [–49–26 60]) in the somatosensory network. The mean BOLD time series were extracted from these seeds. Subsequently, rsFC analysis was performed between the seed and every voxels in the brain. The resulting correlation coefficients were transformed to approximate a Gaussian distribution using Fisher’s *r*-to-*z* transformation.

### Statistical Analysis

Statistical analysis of the rsFC was performed in SPM8 for both seed-based rsFC and ICA. First, the whole brain GMV, years of education and gender were regressed as the potential confounding covariates in the general linear model for each group to correct for the effects of atrophy, education and gender on subsequent rsFC analysis. Then, the within-group *Z*-values maps were analyzed with a random effect one-sample *t*-test. Statistical maps of significant connections with each seed were created for each group. A threshold of *P* < 0.05 (false discovery rate corrected, cluster size >23 adjacent voxels (621 mm^3^) was set to identify the significant level. Second, a two-sample *t*-test was performed with an explicit mask from the union set of the one-sample *t*-test results of the two groups. The significance threshold of group differences was set to *P* < 0.05 (false discovery rate corrected) and cluster size >23 adjacent voxels (621 mm^3^) in the mask.

### Connectome-Based Prediction Framework

To investigate the underlying relationship between altered functional properties in the SM system and age in older adults, we used machine learning algorithms in this study. The leave-one-out-cross-validation (LOOCV) strategy was used to estimate prediction accuracies (Lachenbruch and Mickey, 1968). Prediction process consisted of two steps: training and testing. In the training step, each older adult were designated the test sample in turns while the remaining samples were used to train the predictor model. The altered rsFCs (false discovery rate corrected *P* < 0.05, cluster size >23), which resulted from ICA and seed-based analysis in the training step, were used as features. Then, in the testing step, we predicted the ages of remaining older adult using the same feature. To predict the age of older adults from the local and global change in the SM system, we conducted two-aspect connectome-based prediction frameworks using MVPA on all altered rsFCs and UVPA on single altered rsFCs, respectively. Specific, the MVPA is based on support vector machine (SVM), and UVPA is a machine learning approach combines LOOCV with linear regression.

#### Multivariate Variable Pattern Prediction Analysis

In this study, a support vector regression (SVR) procedure (Smola and Scholkopf, 2004) was used to derive a brain aging of older adults from multivariate pattern. SVR is a supervised learning technique based on the concept of SVM in order to make real-valued predictions. We used the 𝜀–SVR algorithm implemented in LIBSVM (Chang and Lin, 2011) to calculate the regression model used for estimating the brain aging of older adults. To achieve generalized performance, SVR attempts to minimize the training error within the 𝜀 tolerance and the complexity of the regressor (Smola and Scholkopf, 2004). A linear kernel SVR was used in this study. The epsilon parameter was set to its default value, 𝜀 = 0.001. During LOOCV, each older adult was designated the test sample in turns while the remaining samples were used to train the SVR predictor; the trained regression model is used to predict the testing example.

In detail, to improve the performance of the predictor, we first selected the features and then evaluated the age predictions using two nested stratified LOOCV loops (Ambroise and McLachlan, 2002; Huttunen et al., 2012). The features were selected in the inner LOOCV loop and the age predictions were evaluated in the outer LOOCV loop thus avoiding the problem of training on testing data. For each inner LOOCV loop, the correlation coefficient of each feature with the chronological age was computed on the data that is the training set of the outer LOOCV loop. The features were then separately ranked by the absolute value of the correlation coefficients in descending order. The model goodness criterion, which was the number of the ranked features that used in the outer LOOCV loop, was the correlation coefficient (*r*) between the chronological and estimated age. The ranked features, which could get the highest *r* between the chronological and estimated age, were retained, while the rest were eliminated. Since features ranking was based on a different subset of data for each of inner LOOCV, the selected features was slightly different among results of each inner LOOCV. The consensus features were selected to form part of the predictor.

#### Univariate Pattern Prediction Analysis

We also conducted connectome-based prediction frameworks from univariate pattern based on single feature that was the altered rsFC in the older compared with younger adults in the training step. Here, a machine learning approach with LOOCV was used together with linear regression (Cohen et al., 2010). The age variable for older adults was referred to as “label”. LOOCV was performed with this label. The dependent variable (age of older adults) and the independent variable (averaged value) were inputted into a linear regression algorithm. A linear regression model was established using altered rsFC chosen from the training step. Predicted values were obtained for the remaining older adult. This procedure was repeated to obtain a final result. The technical details are provided in Supplemental Information (see Results).

#### Model Prediction Evaluation

The two-aspect (MVPA and UVPA) models’ accuracy in predicting older adults’ age according to altered rsFCs were evaluated using two statistical measures. First, Pearson correlation coefficient [r_(predicted, observed)_] was computed between chronological and estimated age. A nonparametric testing approach was used to test the null hypothesis of no significant correlation. The chronological ages were randomly permuted 1000 times, and the entire prediction process was carried out with each one of the randomized prediction labels. The statistical significance (*p*-values) of the permutation test represent the probability of observing the reported accuracy by chance [(number of permutation *r*_(predicted, observed)_ < observed r_(predicted, observed)_) + 1)/(number of permutations + 1)]. Only an extent threshold *p* < 0.05 is reported. Second, the mean absolute error (MAE) which measures the average magnitude of errors between chronological age and model predicted age was calculated. Low MAE value means better prediction than high MAE value.

### Validation: Reproducibility

There is currently no consensus over whether the whole brain signal should be removed in the preprocessing of the resting-state fMRI data. The global signal is confounded with physiological noise, which has been reported by several studies (Birn et al., 2006), and should be removed (Fox et al., 2009). On the other hand, other studies have suggested that global signal regression (GSR) could introduce negative rsFC (Murphy et al., 2009; Weissenbacher et al., 2009), and is associated with the neuronal signal (Schölvinck et al., 2010). To ensure that the results were not outcome of GSR, we constructed the resting state networks of the primary motor somatosensory cortices using a seed-based rsFC analysis without GSR. Then, we also recomputed the prediction analysis, which included two-aspect connectome-based frameworks from MVPA and UVPA tools, based on the altered rsFCs resulted from GSR.

We further added the GMV as a control feature, combined with all altered rsFC features, decreased rsFC features of insula, as well as increased rsFC features, respectively, in the UVPA and MVPA tools to compare the prediction contribution of the increased and decreased features in the altered SM system of older adults.

## Results

### Participant Fundamental Information

Sixteen older adults were excluded because of low MoCA score (five subjects), poor performance on the SF-36 (three subjects), and excessive head motion (eight subjects). Six younger adults were also excluded because of excessive head motion. Thus, 52 older subjects [age (mean ± SD): 63.2 ± 5.8 years (51–76 years), *n* = 30 females] and 51 younger subjects [age: 18–26 years (21.5 ± 1.9 years), *n* = 26 females] were included in further rsFC analysis. In addition, we compared the *FD_translation_* and *FD_rotation_* values between the remained subjects of two groups to evaluate the homogeneity of head motion between two groups. There were no significant differences between the two groups concerning FD values (two-sample two-tailed *t*-tests, *T =* 1.02*, P =* 0.31 for *FD_translation_*, and *T =* 1.20*, P =* 0.23 for *FD_rotation_*). There also were no significant differences between the two groups in gender (Chi square test, *P =* 0.49). Younger adults had more years of education compared with older adults (two-sample two-tailed *t*-tests, *T =* 9.35, *P* < 0.001). Compared with younger adults, significantly decreased whole GMV was found in older adults (two-tailed *t*-test, *T* = 4.32, *P* < 0.001).

### Analysis of Sensorimotor Network from ICA Analysis

**FIGURE 1:**
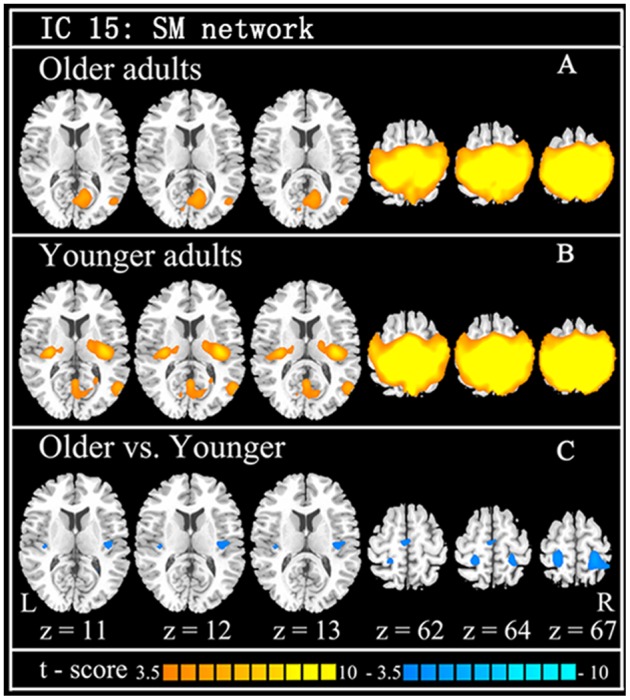
**The findings of functional connection of SM network from ICA.** Independent component (IC) of SM network in older adults (**A**: the first row) and younger adults (**B**: the second row), and the between-group difference (**C**: the last row) are demonstrated. Cool color indicates decreased functional connections when older adults compare to younger adults. For display purposes, all of the maps are shown with *t* score between ±3.5 and ±10.

Using GICA, 36 components were estimated by MDL criterion (Li et al., 2007), which include default mode network, auditory network, sensorimotor network, visual network, cerebellum network, and frontal-parietal network, for both groups. Because this study focused on SM system, the independent component (IC 15) including the supplementary motor area, sensorimotor cortex, and secondary somatosensory cortex, was selected as SM network, which is consistent with previous results (Smith et al., 2009). Compared with the younger adults, the older group showed the significantly decreased functional connections among the main regions in the SM network, including SMA, pre/postcentral, superior parietal lobule, mid-posterior insula, and rolandic operculum (**Table 1**; **Figure 1**).

**Table 1:** Significantly decreased functional connections among the SM network in older adults compared with younger adults.

		MNI coordinates				
Regions	BA	*x*	*y*	*z*	Peak *T*-score	Cluster voxels
**Younger > older**						
Left postcentral	BA 4	–24	–34	72	4.71	97
Left superior parietal	BA 7	–26	–47	69	3.93	
Right postcentral	BA 4	22	–35	71	6.09	184
Right superior parietal	BA 5	18	–49	68	5.03	
Right precentral	BA 6	20	–26	71	4.68	
Left insula	BA 48	–35	–19	15	4.12	24
Left rolandic operculum	BA 48	–41	–21	15	3.76	
Right insula	BA 48	37	–16	13	4.47	37

*BA, Brodmann area.*

### Seed Based Functional Connectivity Analysis

**FIGURE 2:**
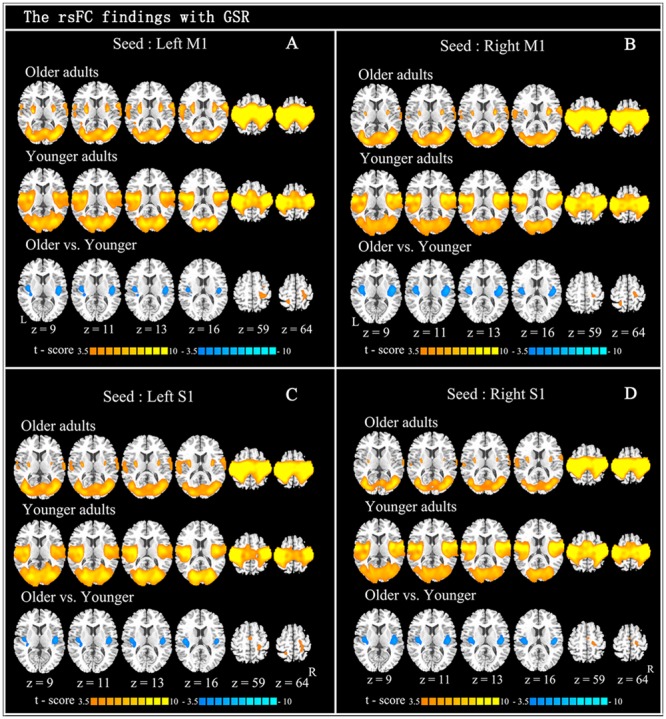
**Resting-state functional connection findings with global signal regression (GSR).** Patterns of significant positive correlation with four seeds: left M1 (**A**: *x* = –44, *y* = –15, *z* = 58), right M1 (**B**: *x* = 47, *y* = –15, *z* = 57), left S1 (**C**: *x* = –49, *y* = –26, *z* = 60), and right S1 (**D**: *x* = 53, *y* = –26, *z* = 59), in older adults (the first row) and younger adults (the second row), and the between-group difference (the last row) are demonstrated. Cool color indicates decreased functional connections and hot color indicates increased functional connections, when older adults are compared to younger adults. For display purposes, all of the maps are shown with *t* score between ±3.5 and ±10.

**FIGURE 3:**
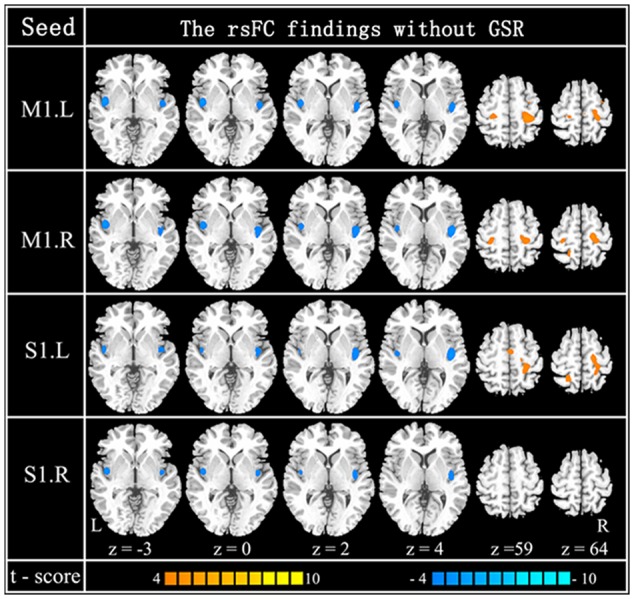
**Resting-state functional connection findings without GSR.** Patterns of between-group difference of four RSNs using four seeds: left M1 (*x* = –44, *y* = –15, *z* = 58) (the first row), right M1 (*x* = 47, *y* = –15, *z* = 57) (the second row), left S1 (*x* = –49, *y* = –26, *z* = 60) (the third row) and right S1 (*x* = 53, *y* = –26, *z* = 59) (the last row). Cool color indicates decreased functional connections and hot color indicates increased functional connections, when older adults are compared to younger adults [*P* < 0.0001 (uncorrected) and cluster size >23 adjacent voxels (621 mm^3^)].

The within-group rsFC maps were generated for each group. In the younger adults, the bilateral M1 was positively correlated with the pre- and postcentral gyrus, middle occipital gyrus, superior temporal gyrus, SMA, putamen, and insula (**Figures 2A,B**). In the older adults, the bilateral M1 was positively correlated with similar brain regions such as in younger adults (**Figures 2A,B**). Relative to the younger adults, the older adults showed significantly increased rsFC seeded at bilateral M1 to pre- and postcentral gyrus and superior parietal lobule, while decreased rsFC was detected in the bilateral insula and rolandic regions (**Table 2**; **Figures 2A,B**). In the younger adults, the signals from pre- and postcentral gyrus, superior frontal gyrus, SMA, and insula were positively correlated with the signals from bilateral S1 (**Figures 2C,D**). In the older adults, the bilateral S1 were positively correlated with similar brain regions to those of the younger adults (**Figures 2C,D**). Compared to the younger adults, significantly increased connections were observed among the primary sensorimotor cortex and superior parietal lobule, while decreased connections were detected in the bilateral insula and rolandic regions (**Table 2**; **Figures 2C,D**). These results were largely preserved after accounting for the effects of global signal removal (**Figure 3**; Supplementary Table S1). Other details are provided in Supplemental Information (see Intrinsic Functional Connectivity Without Global Signal Regression Analysis).

**Table 2:** Significant differences for resting-state functional connections with bilateral M1 and S1 in older adults compared with younger adults.

		MNI coordinates				
Regions	BA	*x*	*y*	*z*	Peak *T*-score	Cluster voxels
**Left M1**						
**Younger < older**						
Right postcentral	BA 3	23	–38	64	4.38	86
Right precentral	BA 6	20	–22	65	3.91	
Left superior parietal	BA 2	–21	–47	64	4.28	42
**Younger > older**						
Right insula	BA 48	40	–13	10	5.32	229
Right rolandic operculum	BA 48	44	–16	17	5.03	
Right superior temporal	BA 48	48	–19	4	4.83	
Left insula	BA 48	–39	–11	9	4.95	139
Left rolandic operculum	BA 48	–43	–17	18	4.38	
Left superior temporal	BA 48	–49	–5	0	3.93	
**Right M1**						
**Younger < older**						
Left superior parietal	BA 5	–21	–49	67	3.67	33
**Younger > older**						
Right insula	BA 48	39	–12	10	6.28	319
Right rolandic operculum	BA 48	44	–16	17	5.47	
Right superior temporal	BA 48	46	–16	3	3.97	
Left insula	BA 48	–39	–13	10	5.67	302
Left rolandic operculum	BA 48	–42	–17	17	5.22	
Left superior temporal	BA 48	–45	–24	7	4.23	
**Left S1**						
**Younger < older**						
Right precentral	BA 6	22	–27	65	3.86	66
Right postcentral	BA 2	24	–42	63	3.65	
Right supplementary motor area	BA 6	–2	–10	57	4.03	23
Left supplementary motor area	BA 6	2	–10	55	3.94	
Left superior parietal	BA 5	–18	–52	67	3.99	39
**Younger > older**						
Right insula	BA 48	40	–13	9	5.22	175
Right rolandic operculum	BA 48	44	–14	17	5.08	
Right superior temporal	BA 48	46	–16	4	4.31	
Left insula	BA 48	–39	–10	9	4.99	164
Left rolandic operculum	BA 48	–43	–19	16	4.92	
**Right S1**						
**Younger < older**						
Right precentral	BA 6	19	–26	65	3.72	36
**Younger > older**						
Right insula	BA 48	42	–13	8	5.57	249
Right rolandic operculum	BA 48	41	–18	19	4.51	
Right superior temporal	BA 48	49	–22	6	3.81	
Left insula	BA 48	–36	–11	10	4.83	253
Left rolandic operculum	BA 48	–44	–18	17	4.61	
Left superior temporal	BA 48	–47	–19	6	3.61	

*BA, Brodmann area*

**FIGURE 4:**
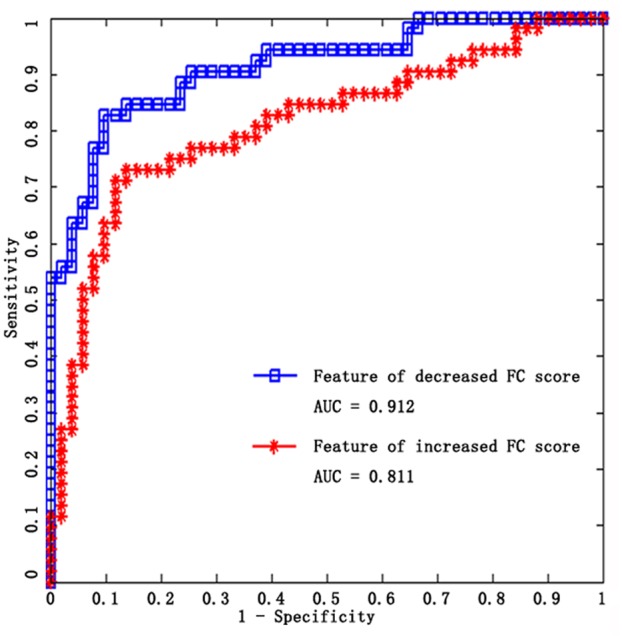
**ROC curves of the two SVM classifiers, blue for decreased rsFC feature, red for increased rsFC feature**.

To compare the contribution of the significantly increased and decreased rsFC in the altered SM system of older adults, MVPA was used in this study, since the contribution would be positively related with the performance of classifier (the detailed processing see Section “Comparison between Increased rsFC and Decreased rsFC through Multivariate Classification” in Supplemental Information). SVM classifiers were adopted here to classify older adults from younger adults using increased functional connections and decreased functional connections as features, respectively. Results show that linear SVM classifier with decreased rsFC score feature performs better than linear SVM classifier with increased rsFC score feature in terms of accuracy, sensitivity, specificity, and AUC value (**Table 3**; **Figure 4**). Other details are provided in Supplemental Information (see Comparison between Increased rsFC and Decreased rsFC through Multivariate Classification).

**Table 3:** Classification performance for SVM classifier based on increased rsFC score and decreased rsFC score, respectively.

SVM classifier	Accuracy	Sensitivity	Specificity	AUC	*P*
Decreased sFC feature	83.50%	84.62%	82.35%	91.21%	*P* < 0.001
Increased rsFC feature	71.84%	71.15%	72.55%	81.15%	*P* < 0.001

### Prediction of Older Adult’s Chronological Age

We further examined the intrinsic functional connectivity of the sensorimotor system in relation to age in the older adults. According to the differences between groups in the training step, regions with significantly altered rsFC were chosen for the following machine learning prediction analysis: bilateral mid-posterior insula, superior parietal lobule, SMA, and pre/postcentral resulted from seed-based rsFC comparison, superior parietal lobule, mid-posterior insula, SMA and postcentral resulting from ICA comparison.

**FIGURE 5:**
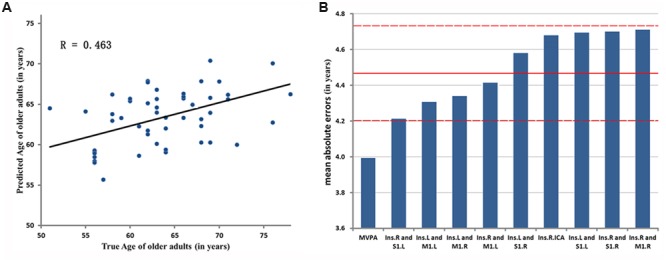
**Evaluation of two-aspect connectome-based prediction framework. (A)** Denotes the prediction results based on MVPA. The chronological age is shown in *x*-axis, and estimated age in *y*-axis. *R* denotes correlation coefficient between chronological and estimated age. **(B)** Represents Mean Absolute Errors (MAEs) between the estimated and chronological age based on MVPA and UVPA, respectively. The features of MVPA and UVPA are shown in *x*-axis, respectively, and the value of MAE in *y*-axis. ‘Ins.R and M1.L’ represents the univariate feature that is the rsFC between right mid-posterior insula and left M1. ‘Ins.R.ICA’ denotes the univariate feature resulted from ICA analysis. The red lines denote the mean ± SD values (4.471 ± 0.25) of MAE.

The result of MVPA [*r*_(predicted, observed)_ = 0.463, *p* < 0.001, MAE = 3.993, **Figure 5**] represents that the age of older adults could be predicted through the features which come from fifty altered rsFC features. Five consensus features (left insula and left M1, left insula and right M1, left insula and right S1, right insula and left M1, right insula and left S1), which were used in the outer LOOCV loop, were observed. Furthermore, the univariate pattern connectome-based prediction analysis also revealed that, in older adults, age could be reliably predicted by the decreased rsFC value in the right mid-posterior insula resulting from ICA analysis [*r*_(predicted, observed)_ = 0.237, *p* = 0.026, MAE = 4.698, **Figure 5**), as well as through decreased rsFC values between sensorimotor cortex and bilateral mid-posterior insula (**Table 4**; **Figure 5**).

**Table 4:** Resting-state functional connectivity (rsFC) predicts the age of older adults.

		Prediction analysis		
Prediction framework	rsFC	*r*_(predicted, observed)_	*P*	MAE
UVPA	Ins.L and S1.L	0.311	0.041	4.704
UVPA	Ins.L and S1.R	0.326	0.015	4.591
UVPA	Ins.L and M1.L	0.401	0.006	4.317
UVPA	Ins.L and M1.R	0.381	0.036	4.336
UVPA	Ins.R and S1.L	0.400	0.013	4.218
UVPA	Ins.R and S1.R	0.304	0.022	4.716
UVPA	Ins.R and M1.L	0.383	0.030	4.408
UVPA	Ins.R and M1.R	0.297	0.046	4.726

*UVPA, univariate pattern analysis; rsFC, resting-state functional connectivity; Ins, insula; MAE, mean absolute error*

In addition, these results were also largely preserved after accounting for the effects of global signal removal (Supplementary Table S2; Supplementary Figure S1). More details are provided in Supplemental Information (see Detailed UVPA Prediction Steps and Results). The UVPA results resulted from features, which are not significant through permutation test, are provided in Supplemental Information (see Materials; Supplementary Tables S3.1–S3.3). Finally, the prediction analyses, which are based on different sets of features, show that the prediction results with insular features performs better than other sets of features. Other details are provided in Supplemental Information (see The Prediction Results Based on Different Sets of Feature; Supplementary Tables S4.1,S4.2).

## Discussion

Our results demonstrated that normal aging is associated with declining functional integration in the primary SM system using resting-state fMRI, and the individual age of older adults can be reliably predicted by the intrinsic functional connectivity of mid-posterior insula through both MVPA and UVPA. The primary SM system was identified and evaluated in terms of two common approaches: ICA and seed-based rsFC analysis. The findings resulting from these two methods revealed robust age effects, indicating that decreases in primary SM system integration correspond with increasing age. In contrast to the declining function of the primary SM system, increased rsFC among primary sensorimotor regions were also found through seed-based rsFC analysis, which revealed that older adults might need a higher degree of anticipatory preparation for the declining sensorimotor function (Mathys et al., 2014; Song et al., 2014). These changes in rsFC might reflect a remodeling of function of the SM system with aging. These findings suggest that the functional connectivity of mid-posterior insula is modified with aging. These findings might provide further insight into changes in primary sensorimotor function underlying rest activity with aging.

The altered functional property of mid-posterior insula in primary SM system observed here is strikingly similar to previous findings. With increasing age, the reduced rsFC between SMA and central insula (Hoffstaedter et al., 2014), cerebellar seed and insula (Seidler et al., 2015), as well as posterior insula and SMA and other sensorimotor regions (Roski et al., 2013) may be associated with general impairments in somatosensory processing in older adults. In the present study, the significantly decreased rsFC of the mid-posterior insula was observed in both ICA and seed-based rsFC analysis in older adults relative to younger adults. The human insula cortex forms a distinct lobe and involves three major functionally distinct sub-regions (Chang et al., 2013). As one of the three sub-regions, the mid-posterior insula region is associated with sensorimotor processing (Stephani et al., 2011). The mid-posterior insula, a more high-level region in sensorimotor processing than the primary sensorimotor cortex, plays an important role in sensorimotor integration processing (Kurth et al., 2010; Nieuwenhuys, 2012; Chang et al., 2013). The mid-posterior insula has also been ascribed an integrative role, linking information from diverse sensorimotor functional regions and playing an important role in sensorimotor processing (Nieuwenhuys, 2012; Chang et al., 2013). Altogether, the key nodes of the SM network, the bilateral mid-posterior insula, showed strongly reduced rsFC in older adults. These findings might reflect that older adults loosened the integration of sensorimotor processing and indicate a reduced ability to modulate activity in the appropriate region of the sensorimotor system. In addition, functional differentiation of the insula cortex was already indicated by recent excellent studies (Nieuwenhuys, 2012; Chang et al., 2013). It is thought to play a role in functional integration between different functional systems by integrating information from diverse functional systems (Nieuwenhuys, 2012). It was reported to be involved in not only processing of the reciprocal influence of emotion and interoception, but also integrating between cognitive tasks and emotion as well as sensation (Critchley, 2005). The decreased rsFC of the insula observed in the current study may influence the interregional integration among attention, emotion or other functional system in the older adults. This speculation was also validated in children. For example, the mid-posterior insula could mediate empathy when children observed a signal indicating others were receiving a pain stimulus by associating it with fronto-parietal attention network (Decety et al., 2008). These findings might be important for the future studies in cognitive disorders and healthy aging.

Furthermore, several previous studies which were either cortical thickness (Rodrigue and Kennedy, 2011; Khundrakpam et al., 2015) or activation fMRI studies (Dosenbach et al., 2010; Qiu et al., 2015) have reported that the SM system contributes to estimate the age of young adults and aging subjects. In this study, we found that the age of older adults could be predicted by decreased rsFC value between the mid-posterior insula and primary sensorimotor cortex, as well as decreased rsFC value of mid-posterior insula resulted from ICA analysis. The functional property of bilateral mid-posterior insula is the exclusive consensus features in the stage of selecting features in MVPA. These analyses revealed that weakening connections of mid-posterior insula contributed more to predicting the age of older adults than other features in SM system. Our findings provide new evidence that functional connectivity of mid-posterior insula in SM system is associated with the individual age of older adults. Interestingly, machine learning approaches revealed that the rsFC of mid-posterior insula in the SM system could also predict the age in older adults.

The primary somatosensory cortex is considered to be the main area of the SM system (Allen et al., 2011). Some previous studies based on ICA have demonstrated that the decreased integration of the SM network may be associated with perceptual impairments in patients with neurological disease (Luo et al., 2011; Li et al., 2015). Our findings from ICA also reflect the declining functional integration in sensorimotor areas in aging. Moreover, the decreased rsFC between the somatosensory cortex and mid-posterior insula was observed through seed-based rsFC analysis. Several recent studies have reported increasing rsFC in SM system with age (Langan et al., 2010; Song et al., 2014; Zhang et al., 2015). Hoffstaedter et al. (2014) reported that each S1/M1 showed age-related decrease of resting state rsFC with primary sensorimotor regions, while right S1/M1 featured age-dependent increase of rsFC with SMA, superior parietal lobule. In several studies, increasing sensorimotor connectivity with age has been suggested to be compensatory (Mathys et al., 2014; Song et al., 2014). We have also documented that increased rsFC was found in some primary SM regions through the seed-based rsFC analysis. Although these results were different with the findings from ICA, both methods could evaluate the impact of healthy aging on the SM system from different aspects (global and local aspect). The observed results from ICA reveal that the declining functional integration (global aspect) was observed in SM system in aging. The findings from seed-based analysis might indicate that the increased rsFC (local aspect) in aging responds to the declining sensorimotor function. Some researchers also found the relationship between increased interhemispheric motor rsFC and reductions in interhemispheric inhibition with age (Fling and Seidler, 2012), suggesting that the increased rsFC may derive from age-related declines in inhibitory neurotransmitters. In addition, the linear SVM classifier with decreased rsFC score feature performs better than linear SVM classifier with increased rsFC score feature. The contribution of significantly changed SM system with decrease functional connectivity is stronger than that with increased functional connectivity.

Noteworthy, rsFC was related with behavior performance outside the MRI scanner (Seidler et al., 2015). Resting state connectivity could be regarded as offering a potential prediction indicator for task performance. Actually, some studies have reported that rsFC provided pre-task brain activation level, which was partly consistent with subsequent task results (Langan et al., 2010; Wang et al., 2010). Specifically, stronger resting state rsFC in hippocampal network might predict better memory task performance (Wang et al., 2010). Our findings of altered rsFC in primary SM system of older adults may be associated with the common decline of motor performance in aging.

## Limitations

While we believe our findings provide a further insight into changes in SM system in the healthy aging brain, there are a number of important caveats in interpreting these results. First, physiological noise should be considered in the rsFC analysis. In the present study we cannot eliminate cardiac and respiratory fluctuations completely through temporal filtering (band-pass 0.01–0.08 Hz). Second, we only delineated trajectories of the aging based on altered rsFC within primary SM system. We could not conclude that the top predictors were highly localized in primary SM system in healthy aging elder adults. The important regions or networks will also be examined in aging through the machine learning framework in future. Third, the current approach investigates the age in a cross-sectional rather than longitudinal fashion. However, we are following this cohort of older adults and will acquire data each year. The progressive effect of aging in the remodeling of rsFC in the SM system will be considered in future studies through a longitudinal analysis. Finally, testing for motor-related function was not included in the current study. Though no significant relationships were observed after we measured the association between the behavior features (the scores of SF-36 and MoCA) and age of older adults and altered rsFCs. Our findings may involve a confusion, in which the declined motor performance in older adults would affect the associations observed here. However, the physical functioning (PF) scores, which were extracted from the SF-36 test, may reflect a health scale about motor-related function to some degree. Thus, the partial correlations between the age of older adults and change rsFCs value were calculated, accounting for the effects of gender, years of education, whole brain GMV, and PF. Likewise, the relationship between the rsFCs of insula and the age of older adults were also found (Supplementary Table S5). More details are provided in Supplemental Information (see Correlations between Functional Properties and the Age of Older Adults Controlling for the Physical Functioning Related with Motor). The PF value was not the comprehensive behavior performance outside the MRI scanner. This defect would be investigated in the future study.

## Conclusion

We analyzed the rsFC changes in the SM system in older adults compared to younger adults, which demonstrated significant remodeling of resting state primary sensorimotor system. The altered rsFC may be suggestive of the loosened integration of sensorimotor processing and might also imply the compensation in the primary sensorimotor network in older adults. Furthermore, we demonstrated that the MVPA and UVPA extract sufficient information from these decreased rsFC to make reliable predictions about individuals’ chronological age across healthy aging. This study may help to investigate the potential reorganization of the SM system in the brain of older adults.

## Ethics Statement

The study was approved by the Ethics Committee of University of Electronic Science and Technology of China in accordance with the Helsinki Declaration. Written informed consent was obtained from each patient and control subject. All the participants were volunteers. They were recruited from the local communities.

## Author Contributions

Conceived and designed the work: HH, CL, BB, MB, DY. Acquired the data: XC, WC, JG, BB. Analyzed the data: HH, CL. Wrote the paper: HH, CL. All authors revised the work for important intellectual content. All of the authors have read and approved the manuscript.

## Conflict of Interest Statement

The authors declare that the research was conducted in the absence of any commercial or financial relationships that could be construed as a potential conflict of interest.

## Footnotes

1 http://www.fil.ion.ucl.ac.uk/spm/

2 http://mialab.mrn.org/software/gift/

3 http://research.ics.tkk.fi./ica/icasso
