# Supplementary material for: The Functional Integration in the Sensory-Motor System Predicts Aging in Healthy Older Adults
Source: Front Aging Neurosci. 2017 Jan 5;8:306. doi: 10.3389/fnagi.2016.00306 (PMC5216620; doi:10.3389/fnagi.2016.00306)
Supplement: Supplementary file 1 [file Presentation_1.pdf]

The functional integration in the sensory-motor system predicts aging in healthy older adults

Supplemental Information

Hui He[1], Cheng Luo[1]*, Xin Chang[1], Yan Shan[1], Weifang Cao[1], Jinnan Gong[1], Benjamin Klugah-Brown[1], Maria Antonieta Bobes[2], Bharat Biswal[3], Dezhong Yao[1]*

1, The Key Laboratory for NeuroInformation of Ministry of Education, Center for Information in Medicine, High-Field Magnetic Resonance Brain Imaging Key Laboratory of Sichuan Province, School of Life Science and Technology, University of Electronic Science and Technology of China, Chengdu, 610054, China

2, Department of Biological Psychiatry, Cuban Neuroscience, Center. Avenida 25, 152002. Cubanacan Playa, La Habana, Cuba

3, Department of Biomedical Engineering, New Jersey Institute of Technology, University Heights, Newark, NJ 07102, USA

*Address correspondence to: Cheng Luo, No. 4, Section 2, North Jianshe Road, Chengdu, P.R. China, 610054. E-mail: chengluo@uestc.edu.cn, Tel: 86 28 61831272; Fax: 86 28 83206978
Dezhong Yao, No. 4, Section 2, North Jianshe Road, Chengdu, P.R. China, 610054. E-mail: dyao@uestc.edu.cn; Tel: 86 28 83201018; Fax: 86 28 83206978.

Short title: Predicts aging through sensorimotor system

Section1. Intrinsic Functional Connectivity without global signal regression analysis

Because of a controversy regarding global signal regression (Murphy et al., 2009), we also constructed the resting state networks of the primary motor somatosensory cortices using a seed-based rsFC analysis without global signal regression. Based on our previous research work, (Luo et al., 2012) four nearly spherical regions (radius 6 mm) were selected as seeds; including the bilateral primary motor cortex (right M1, MNI coordinates [47 - 15 57]; left M1, MNI coordinates [- 44 - 15 58]) for the motor network, and the bilateral primary somatosensory cortex (right S1, MNI coordinates [53 - 26 59]; left S1, MNI coordinates [- 49 - 26 60]) in the somatosensory network. The mean BOLD time series was extracted from these seeds. Subsequently, rsFC analysis was performed between the seed and all voxels in the brain. The resulting correlation coefficients were transformed to approximate a Gaussian distribution using Fisher's r-to-z transformation. In this way, rsFC maps of these seeds were produced for each participant.

Statistical analysis of the rsFC was performed in SPM8. First, the whole brain GMV, gender and the years of education were regressed as the potential confounding covariates in the general linear model for each group. Then, the within-group Z-values map was analyzed with a random effect one-sample t-test. Statistical maps of significant connections with each seed were created for each group. A threshold of $P < 0.05$ (FDR-corrected, $k = 23$ adjacent voxels) was set to identify the significant level. Second, a two-sample t-test was performed with an explicit mask from the union set of the one-sample t-test results of the two groups. The significance threshold of group differences was set to $P < 0.0001$ (uncorrected) and cluster size > 23 adjacent voxels (621 mm^3).

We found that these two data preprocess did not alter the major results in the present study (Table S1).

Table S1 Significantly differences for resting-state functional connections (without global signal regression) with bilateral M1 and S1 in older adults compared with younger adults

regions	BA	MNI coordinates x	y	z	Peak T-score	Cluster voxels
**Left M1**						
**Younger < older**						
Right Postcentral	BA 3	30	-34	55	4.97	92
Right Precentral	BA 6	30	-17	57	4.26	
Left Precentral	BA 3	-28	-36	59	4.58	73
Left Postcentral	BA 3	-25	-35	64	4.39	
**Younger > older**						
Right Insula	BA 48	43	-7	-2	4.51	124
Right Superior Temporal	BA 48	47	-16	3	4.13	
Left Insula	BA 48	41	-6	-2	4.78	85
Left Superior Temporal	BA 48	-49	-6	-4	4.32	
**Right M1**						
**Younger < older**						
Right Postcentral	BA 4	25	-31	65	4.21	85
Right Precentral	BA 6	19	-23	65	4.16	
Left Precentral	BA 6	-31	-27	63	4.41	53
Left Postcentral	BA 3	-35	-33	57	4.19	
**Younger > older**						

Right Insula	BA 48	41	-12	7	5.32	114
Right Superior Temporal	BA 48	46	-17	3	4.16	
Left Insula	BA 48	-41	-2	-3	4.77	86
Left Superior Temporal	BA 48	-47	3	-7	4.29	
**Left S1**						
**Younger < older**						
Right Precentral	BA 6	29	-22	64	4.51	92
Right Postcentral	BA 3	30	-36	62	4.27	
Left Superior Parietal	BA 5	-22	-52	68	4.37	56
**Younger > older**						
Right Insula	BA 48	41	-14	6	4.59	48
Left Insula	BA 48	-35	-19	16	4.46	43
Left Rolandic Operculum	BA 48	-41	-23	20	4.21	
**Right S1**						
**Younger > older**						
Right Insula	BA 48	41	-9	3	4.49	45
Right Superior Temporal	BA 48	49	-14	2	4.25	
Left Insula	BA 48	-43	-7	1	4.33	35
Left Superior Temporal	BA 48	-50	-8	0	4.11	

BA, Brodmann area

Section2. Comparison between increased rsFC and decreased rsFC through multivariate classification

To compare the contribution of the significantly increase and decrease rsFC in the altered SM system of older adults, multivariate pattern analysis was used in this study. Support vector machine (SVM) classifiers were adopted here to classify older adults from younger adults using increased functional connections and decrease functional connections as feature respectively. The averaged correlation coefficient of clusters that illustrated increased and decreased rsFC with sensorimotor seeds was used as classification feature respectively. A leave-one-out cross-validation (LOOCV) strategy was used to estimate classification accuracies (Wee et al., 2011). A binary label was used here for two groups: 1 for older adults and -1 for younger adults. Classification process consists of two steps: training and testing. During the training step, the model of classification was built using all subjects of two groups leaving out one. The SVM finds a decision boundary that separates the examples in the input space using their class labels. Then, this decision model can be used to predict the class label of a new testing example. A linear kernel SVM was used in this study, and it has only one parameter *C* that determines the trade-off between training error minimization and allowing misclassifications. In this study, the SVM classifier was implemented using LIBSVM toolbox (Chang and Lin, 2011), with a default value for parameter *C* (i.e., $C = 1$).

In this study, we used accuracy, sensitivity, and specificity to quantify the performance of two classifiers. In addition, the discriminative score of each testing subject was acquired by the SVM classifier. Taking each subject's discriminative score as a threshold, the receiver operating characteristic (ROC) curve of the classifier was yielded. The area under the ROC curve (AUC) could indicate the power of this classification.

$$\text{Accuracy} = \frac{\text{TP} + \text{TN}}{\text{TP} + \text{FN} + \text{TN} + \text{FP}} \qquad (1)$$

$$\text{Sensitivity} = \frac{\text{TP}}{\text{TP} + \text{FN}} \qquad (2)$$

$$\text{Specificity} = \frac{\text{TN}}{\text{TN} + \text{FP}} \qquad (3)$$

TP is the number of true positives: number of older adults correctly classified; TN is the number of true negatives: number of younger adults correctly classified; FP is the number of false positives: number of younger classified as older; FN is the number of false negatives: number of older classified as younger.

Furthermore, the statistical significance of all LOOCV results was assessed using permutation testing. We estimated the empirical cumulative distribution of the classifier and predictor accuracies under the null hypothesis. In the permutation test, the class labels (e.g. older vs. younger) were randomly permuted 1000 times, then the classification process was carried out with each one of the sets of randomized class labels. The *p* value of the permutation test is defined as a fraction of the number of classifiers that built based on 1000 sets of randomized class labels, which is better than the classifier that built based on original class label.

Result show that linear SVM classifier with decreased rsFC score feature performs better than linear SVM classifier with increase rsFC score feature in terms of accuracy, sensitivity, specificity, and AUC value.

Section3. Detailed UVPA prediction steps and results

A machine learning approach with balanced 4-fold cross-validation combined with linear regression (Cohen et al., 2010) was conducted to examine brain-based predictors of individual differences in healthy aging. Nonparametric testing was used to assess the performance of the regression model in predicting healthy aging. The age variable for older adults was referred to as “label”. Four-fold balanced cross-validation was performed with this label. The dependent variable (age of older adults) and the independent variable (averaged value of functional connectivity) were inputted into a linear regression algorithm. For the machine learning approach, an equal number of older adults were randomly assigned to each of the four folds (Cohen et al., 2010). A linear regression model was established using three folds chosen randomly from the four folds. Predicted values were obtained for the remaining fold. In brief, we used three folds as the training set to obtain the linear regression model, and the remaining one was used as the testing set. This procedure was repeated 4 times to compute a final $r_{(predicted,\ observed)}$ representing the correlation between the observed data and the data predicted by the regression model. The resulting $r_{(predicted,\ observed)}$ is a measure of how well the independent variable predicts the dependent variable. Finally, the statistical significance of the model was assessed using permutation testing. We estimated the empirical cumulative distribution of the $r_{(predicted,\ observed)}$ by generating 1000 surrogate datasets under the null hypothesis. Each surrogate dataset D_i of size equal to the observed dataset was generated by permuting the labels on the observed data points. The $r_{(predicted,\ observed)}$ was computed using the predicted labels using 4-fold balanced cross validation procedure described above and actual labels of D_i. This procedure produces a null distribution of $r_{(predicted,\ observed)}$ for regression model. The statistical significance (*p* value) of the permutation test is defined as a fraction of the number of $r_{(predicted,\ observed)}$ that built based on 1000 sets of randomized class labels, which is better than the $r_{(predicted,\ observed)}$that built based on original class label. In addition, the mean absolute error (MAE) which measures the average magnitude of errors between chronological age and model predicted age was calculated. Low MAE value means better prediction than high MAE value.

Because of a controversy regarding global signal regression (Murphy et al., 2009), we also performed prediction analysis without global signal regression. We used partial correlation analysis and two-aspect connectome-based frameworks from MVPA and UVPA tools.

The result of MVPA ($r_{(predicted,\ observed)} = .416$, $p < .001$, MAE = 4.202, Figure S3) represents that the age of older adults could be predicted through the features which come from fifty altered rsFC features. Six consensus features (left insula and left M1, left insula and right M1, left insula and left S1, left insula and right S1, right insula and left M1, right insula and right M1), which were used in the outer LOOCV loop, were observed. Furthermore, the univariate pattern connectome-based prediction analysis also revealed that, in older adults, age could be reliably predicted by the decreased rsFC values between sensorimotor cortex and bilateral mid-posterior insula respectively (Table S2, Figure S1.).

Table S2 predictive results based on features that come from seed based rsFC without global signal regression

rsFC	Prediction analysis		
	$r_{(predicted, observed)}$	p	MAE
Ins.L and S1.L	0.351	0.006	4.767
Ins.L and S1.R	0.242	0.029	4.951
Ins.L and M1.L	0.287	0.011	4.825
Ins.L and M1.R	0.304	0.041	4.810
Ins.R and S1.L	0.301	0.011	4.904
Ins.R and S1.R	0.191	0.035	4.962
Ins.R and M1.L	0.322	0.008	4.513
Ins.R and M1.R	0.301	0.009	4.879

Ins: insula; MAE: mean absolute error

The UVPA results of features, which are not significant through permutation test, were provided in following tables.

Table S3.1 predictive results based on features that come from seed based rsFC with global signal regression (non-significant results were found)

rsFC	prediction analysis		
	$r_{(predicted, observed)}$	p	MAE
**Left M1 vs.**			
**Younger < older**			
Right Postcentral	0.0869	0.1014	4.9679
Right Precentral	-0.1042	0.5015	5.1241
Left Superior Parietal	0.1631	0.1240	5.2638
**Younger > older**			
Right Rolandic Operculum	0.2594	0.0625	4.9271
Right Superior Temporal	0.1807	0.0624	5.0124
Left Rolandic Operculum	0.2399	0.1608	4.9487
Left Superior Temporal	0.2479	0.0913	5.0670
**Right M1 vs.**			
**Younger < older**			
Left Superior Parietal	-0.1001	0.3566	5.1290
**Younger > older**			
Right Rolandic Operculum	0.2910	0.0801	4.9738
Right Superior Temporal	0.2243	0.0212	4.9347
Left Rolandic Operculum	0.3107	0.0910	4.9249
Left Superior Temporal	0.2015	0.0659	4.9100
**Left S1 vs.**			
**Younger < older**			
Right Precentral	-0.3531	0.9530	5.2561
Right Postcentral	0.0189	0.2168	5.0388
Right Supplementary Motor Area	0.2016	0.0910	5.0426

Left Supplementary Motor Area	0.1006	0.1001	5.1222
Left Superior Parietal	-0.1243	0.3916	5.0276
**Younger > older**			
Right Rolandic Operculum	0.1215	0.0711	5.1348
Right Superior Temporal	0.1845	0.0929	5.0942
Left Rolandic Operculum	0.1321	0.1849	5.1636
**Right S1 vs.**			
**Younger < older**			
Right Precentral	-0.1590	0.6153	5.0142
**Younger > older**			
Right Rolandic Operculum	0.1922	0.0939	5.2016
Right Superior Temporal	0.1996	0.1371	5.1537
Left Rolandic Operculum	0.2141	0.0782	5.1343
Left Superior Temporal	0.2664	0.0867	5.2302

rsFC: resting-state functional connectivity; MAE, mean absolute error

Table S3.2 predictive results resulted from features that come from seed based rsFC without global signal regression (non-significant results were found)

	prediction analysis		
rsFC	$r_{(predicted,\ observed)}$	p	MAE
**Left M1**			
**Younger < older**			
Right Postcentral	-0.0770	0.2321	5.2150
Right Precentral	-0.0111	0.1923	5.0921
Left Precentral	-0.0121	0.1951	5.0097
Left Postcentral	-0.3682	0.8870	5.2247
**Younger > older**			
Right Superior Temporal	-0.0951	0.4186	5.3202
Left Superior Temporal	0.2587	0.1059	5.0067
**Right M1**			
**Younger < older**			
Right Postcentral	-0.3147	0.8610	5.2237
Right Precentral	-0.1921	0.5729	5.2695
Left Precentral	-0.2176	0.6813	5.1296
Left Postcentral	-0.2976	0.6421	5.2428
**Younger > older**			
Right Superior Temporal	0.1131	0.0849	5.2021
Left Superior Temporal	0.1102	0.0958	5.1256
**Left S1**			
**Younger < older**			
Right Precentral	-0.2786	0.8512	5.1471
Right Postcentral	-0.0613	0.5156	5.1136
Left Superior Parietal	0.0522	0.2198	5.2687

**Younger > older**			
Left Rolandic Operculum	-0.1005	0.4236	5.1182
**Right S1**			
**Younger > older**			
Right Superior Temporal	-0.1102	0.4716	5.2036
Left Superior Temporal	0.1340	0.0829	5.4533

rsFC: resting-state functional connectivity; MAE, mean absolute error

Table S3.3 predictive results resulted from features that come from ICA (non-significant results were found)

	prediction analysis		
regions	$r_{(predicted, observed)}$	p	MAE
Left Postcentral	-0.0180	0.2635	5.1036
Left Superior Parietal	0.0058	0.2312	5.1984
Right Postcentral	-0.2347	0.5713	5.0601
Right Superior Parietal	-0.3381	0.6111	5.2718
Right Precentral	-0.2514	0.4112	5.1675
Left insula	-0.2603	0.6912	5.0935
Left Rolandic Operculum	0.1161	0.0749	5.3500

MAE, mean absolute error

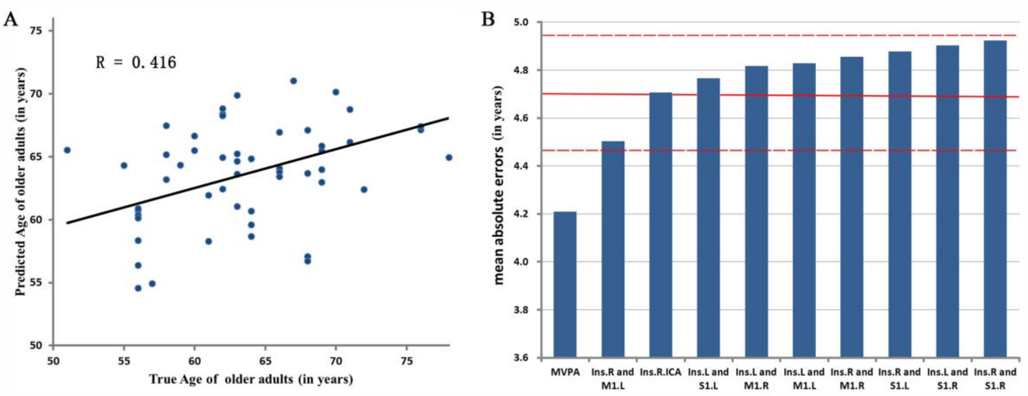

Figure S1 Effect of without global signal regression on the prediction model. A represents the prediction of chronological age based on MVPA. The chronological age is shown in x-axis, and estimated age in y-axis. R denotes correlation coefficient between chronological and estimated age. B denotes Mean Absolute Errors (MAEs) between the estimated and chronological age based on MVPA and UVPA respectively. The features of MVPA and UVPA are shown in x-axis respectively, and the value of MAE in y-axis. 'Ins.R and M1.L' represents the univariate feature that is the rsFC between right mid-posterior insula and left M1. 'Ins.R.ICA' denotes the univariate feature resulted from ICA analysis. The red lines denote the mean ± standard deviation values (4.75 ±0.23) of MAE.

Section4. The prediction results based on different sets of feature

We further added the gray matter volume (GMV) as a control feature, combined with all altered rsFC features, decreased rsFC features, as well as increased rsFC features respectively in the UVPA and MVPA tools to compare the prediction contribution of the increased and decreased features in the altered SM system of older adults. The prediction results represent that the prediction results with insular feature performs better than other sets of features (Table S4.1 and Table S4.2).

Table S4.1 Predictive results based on different sets of features (rsFC with global signal regression)

	UVPA results			MVPA results		
Feature	$r_{(predicted, observed)}$	p	MAE	$r_{(predicted, observed)}$	p	MAE
Decreased insular feature	0.4068	< 0.001	4.5032	0.4123	< 0.001	4.6509
Increased rsFC feature	0.1079	0.0729	5.3234	0.1090	0.1638	5.2051
All altered rsFC feature	0.3355	0.0100	4.6978	0.3024	0.0051	4.5347
Decreased insular feature$^{\#}$	0.3602	0.0170	4.5163	0.3302	0.0090	4.5039
Increased rsFC feature$^{\#}$	0.2172	0.0546	4.7214	0.2151	0.0133	5.0107
All altered rsFC feature$^{\#}$	0.3042	0.0050	4.7936	0.2615	0.0110	4.8911

MAE: mean absolute error; ‘#’ represent that we add the GVM as a control feature in this set feature.

Table S4.2 Predictive results based on different sets of features (rsFC without global signal regression)

	UVPA results			MVPA results		
Feature	$r_{(predicted, observed)}$	p	MAE	$r_{(predicted, observed)}$	p	MAE
Decreased insular feature	0.3815	< 0.001	4.7283	0.3922	< 0.001	4.6336
Increased rsFC feature	0.0909	0.4117	5.3578	0.1163	0.0820	5.2626
All altered rsFC feature	0.3158	0.0080	4.8013	0.3050	0.0070	4.6093
Decreased insular feature$^{\#}$	0.3014	0.0050	4.6356	0.3181	0.0060	4.6024
Increased rsFC feature$^{\#}$	0.0943	0.1185	5.1652	0.1004	0.0770	5.1137
All altered rsFC feature$^{\#}$	0.2951	0.0091	4.9126	0.3033	0.0100	4.8298

MAE: mean absolute error; ‘#’ represent that we add the GVM as a control feature in this set feature.

Section5. Correlations between functional properties and the age of older adults controlling for the physical functioning related with motor

To investigate the underlying relationship between altered functional properties in the SM system and age in older adults, we used partial correlation analysis in this study. Testing for motor-related function was not included in the current study. First, we extracted the average correlation coefficient (CC) of regions that illustrated different rsFCs with sensorimotor seeds, as well as the mean CC of the regions of independent components, where differences between groups were observed by comparing the SM network resulting from ICA. Then, the partial correlations between those averaged values and age were calculated, respectively, accounting for the effects of gender, years of education, whole brain GMV and physical functioning (PF). PF scores, which were extracted from the SF-36 test, may reflect a health scale about motor-related function to some degree.

Partial correlation analysis revealed that, in older adults, age has a negative relationship with mean rsFC value (correlation coefficient) between the primary sensorimotor cortex and decreased rsFC brain regions (bilateral insula) (Table S5 A). In addition, these results were also largely preserved after accounting for the effects of global signal removal (Table S5 B). We also found that the mean rsFC value in right insula with decreased rsFC resulted from ICA analysis was negatively correlated with age (r = -.365 p = .009). In general, the results, which demonstrated the relationship between the rsFCs of insula and the age of older adults controlling for the PF scores, were similar to the findings showed in the Table 4 in main text.

Table S5 Correlations between rsFCs of mid-poster insula and the age of older adults

rsFC	A: Partial correlation analysis		B: Partial correlation analysis	
	r	p	r	p
Ins.L and S1.L	-0.239	0.097	-0.278	0.053
Ins.L and S1.R	-0.295*	0.039*	-0.271	0.059
Ins.L and M1.L	-0.334*	0.018*	-0.369*	0.009*
Ins.L and M1.R	-0.234	0.104	-0.281	0.051
Ins.R and S1.L	-0.341*	0.016*	-0.394*	0.005*
Ins.R and S1.R	-0.288*	0.044*	-0.381*	0.007*
Ins.R and M1.L	-0.340*	0.016*	-0.327*	0.021*
Ins.R and M1.R	-0.266	0.064	-0.349*	0.013*

Ins: insula; 'A' represents the relationship with the global signal regression analysis; 'B' represents the relationship after accounting for the effects of global signal removal; '*' represents the significant relationship between two variates (uncorrected $p < 0.05$).

Supplementary References:

Chang, C.C., and Lin, C.J. (2011). LIBSVM: A Library for Support Vector Machines. *Acm Transactions on Intelligent Systems and Technology* 2(3). doi: Artn 27

Doi 10.1145/1961189.1961199.

Cohen, J.R., Asarnow, R.F., Sabb, F.W., Bilder, R.M., Bookheimer, S.Y., Knowlton, B.J., et al. (2010). Decoding developmental differences and individual variability in response inhibition through predictive analyses across individuals. *Front Hum Neurosci* 4**,** 47. doi: 10.3389/fnhum.2010.00047.

Luo, C., Guo, Z.W., Lai, Y.X., Liao, W., Liu, Q., Kendrick, K.M., et al. (2012). Musical training induces functional plasticity in perceptual and motor networks: insights from resting-state FMRI. *PLoS One* 7(5)**,** e36568. doi: 10.1371/journal.pone.0036568.

Murphy, K., Birn, R.M., Handwerker, D.A., Jones, T.B., and Bandettini, P.A. (2009). The impact of global signal regression on resting state correlations: are anti-correlated networks introduced? *Neuroimage* 44(3)**,** 893-905. doi: 10.1016/j.neuroimage.2008.09.036.

Wee, C.Y., Yap, P.T., Li, W., Denny, K., Browndyke, J.N., Potter, G.G., et al. (2011). Enriched white matter connectivity networks for accurate identification of MCI patients. *Neuroimage* 54(3)**,** 1812-1822. doi: 10.1016/j.neuroimage.2010.10.026.
